# Depression and suicidality among adolescents living with human immunodeficiency virus in Lagos, Nigeria

**DOI:** 10.1186/s13034-020-00337-3

**Published:** 2020-08-14

**Authors:** Suraju Adeyemo, Increase I. Adeosun, Oluwayemi Cecilia Ogun, Abiodun Adewuya, Agatha Nkiru David, Abosede Adekeji Adegbohun, Olurotimi Adejumo, Oyewale Alfred Ogunlowo, Oluwatosin Oluwaseun Adeyemo

**Affiliations:** 1 Synapse Services, Number 60 Babapomile Street, Onipetesi Estate, Mangoro, Lagos, Nigeria; 2 Department of Medicine Benjamin Carson Snr. School of Medicine, Babcock University, Ilishan-Remo, Nigeria; 3 Federal Neuropsychiatric Hospital, Yaba, Lagos, Nigeria; 4 Department of Behavioral Medicine, Lagos State University College of Medicine, Lagos, Nigeria; 5 Nigeria Institute of Medical Research, Yaba, Lagos, Nigeria; 6 University College Hospital Ibadan, Lagos, Nigeria; 7 Department of Hematology, Lagos State University College of Medicine, Lagos, Nigeria

**Keywords:** Adolescents, Depression, Suicidality, HIV, Nigeria

## Abstract

**Background:**

Nigeria is considered to have the second highest number of people living with human immunodeficiency virus (HIV) worldwide with a national HIV infection prevalence of 5.2% in children and adolescents. Adolescents with HIV-infection have been reported to be more prone to developing comorbid emotional difficulties including depression and suicidality compared to those without HIV-infection. This study is aimed at determining the prevalence and correlates of depression and suicidality in adolescents living with HIV infection.

**Methods:**

Through a consecutive sampling method, two hundred and one adolescents attending HIV outpatient clinics in two tertiary hospital (Lagos state University Teaching Hospital and Nigerian Institute of Medical Research) were recruited. Confidentiality was assured and maintained. Suicidality and Depression were assessed with their corresponding modules in Mini International Neuropsychiatric Interview for children and adolescents (MINI-Kid) by researcher, while the independent variables were assessed using self-administered questionnaires. Data was analyzed with Statistical Package for Social Science version 20.

**Result:**

The prevalence of current and lifetime major depressive episode, and suicidality were 16.9%, 44.8% and 35.3% respectively. Female gender, decreased cluster of differentiation 4 (CD4) count and high adverse childhood experience (ACE), were significantly associated with current depressive episode, while poor social support, high ACE, physical abuse, contacting HIV infection after birth and disclosure of status, were associated with lifetime major depressive episode. Factors associated with suicidality were high ACE score, physical abuse, and emotional abuse. After logistic regression analysis; gender, high ACE and CD4 level were independently associated with current major depression, while only poor social support and contracting HIV infection after birth, were independently associated with lifetime major depression. There was a positive correlation between suicidality and depression.

**Conclusion:**

The presence of high rate of depression and suicidality among adolescents living with HIV-infection in the current study clearly shows the need for regular psychological assessment in these group of adolescents, and thus a strong indication for a multidisciplinary management in them.

## Background

The Human Immunodeficiency virus (HIV) infection is a global pandemic and a major public health problem. HIV infection has claimed about 25 million lives around the world since its discovery about 35 years ago [1]. According to the World Health Organization, about 35 million people are affected by HIV including more than one million children, with between 940,000 and 1.3 million people’s lives lost to HIV infection complication in 2015, 250,000–430,000 of which were from west Africa [2].

Nigeria with the population size of about 160 million is considered to have the second highest number of people living with HIV (PLWH) worldwide (3.2 million) after South Africa (5.6 million). Nigeria has been reported to have a national HIV prevalence of 2.9% in adults and 5.2% in adolescents, with adolescent girls being three times more vulnerable than boys [2].

Adolescents with HIV infection are faced with developmental challenges of this stage, such as puberty, sexuality and the desire to “fit in” which may be seriously complicated by HIV disease. They are also confronted with the challenges of the need for lifelong treatment, education and career progression [3]. They are thus prone to comorbid mental illness such as depression and suicidality [4, 5].

The prevalence of depression among general population of adolescents was 8% worldwide, while the prevalence in developing countries ranged between 2 and 8% [6]. Prevalence of depression has been reported to be higher in adolescents with HIV compared to adolescents who are seronegative worldwide, with a rate of between 20 and 40% in adolescents living with HIV [7]. While suicidality has been extensively studied in the general and medical population, its relationship with HIV infection is under-researched, especially in adolescents [8].

Several risk factors have been reported to be associated with depression and suicidality in patients living with HIV. Some of the factors reported in Africa include younger age, female gender, psychiatric disorders, poor social support, fear of disclosure, stigmatization, socio-economic pressure and cognitive deficits [9, 10]. An indirect relationship has been shown to exist between CD4 count and depression. As individuals with low CD4 count tend to have reduced immunity, higher incidence of opportunistic infection and hospital admission. This leads to deterioration in their overall quality of life, which may then increase their vulnerability to depression and suicidality [11–13].

Depression and suicidality are important co-morbidities in HIV-positive adolescent, and they have been linked to faster disease progression and increased morbidity and mortality. Failure to identify and address these issues may prevent adolescents from successfully coping with their illness or adhering to antiretroviral treatment [14, 15]. Despite this, there was deficiency of knowledge on the prevalence and psychosocial characteristics of suicidality and depression among adolescent with HIV infection in Nigeria as most previous studies in this region had focused on Adult and a few on children with HIV infection [5, 9].

The findings from this study will give information on the prevalence and correlates of depression and suicidality in adolescents with HIV in Nigeria. This may serve as a guide for future research and assist in the formulation of preventive and interventional strategies so as to reduce the occurrence of the disorder in these adolescents.

## Methodology

Setting: This study was conducted between January and July 2016, in 3 facilities including Hematology out-patient clinic and Pediatrics outpatient clinic, both of Lagos State University Teaching Hospital (LASUTH), and Adolescents outpatient HIV clinic of Nigerian Institute of Medical Research (NIMR) Yaba, Lagos. The 3 clinics have a total of about 400 registered adolescents (Hematology outpatient-80, Pediatrics clinic-120 and Adolescents outpatient HIV clinic-200). There is a combined 5 clinic days for the 3 facilities per week (Hematology—3 clinics, Pediatric and Adolescents HIV—1 clinic each).

Participants: The participants for this study included adolescents within ages 10 and 19 years recruited from either of the clinics with a laboratory confirmed diagnosis of HIV, who gave assent to participate in the study in addition to their caregivers’ consent. Excluded are adolescents with other co morbid chronic medical conditions (like Sickle Cell Disease, Diabetes, leukemia etc.) and those that were acutely too ill to be interviewed.

Study design: The study was a cross sectional study.

Study instruments: Data was collected using the following

1. *A socio*-*demographic* questionnaire detailing (a) child variables such as age, gender, ethnicity, class in school; (b) family variables including parent’s marital status, who the primary care giver is, family type (monogamous/polygamous),
2. *Illness variables* such as sources of infection, age at diagnosis, history of hospital admission on account of HIV, history of loss of family member due to HIV-AIDS.
3. *Social support* was assessed using the “OSLO-3 item social support scale”. The OSLO-3 is a brief questionnaire that itemizes support from friend/neighbors, number of close friends/relations and concern shown by people around. The first 2 questions have options (scored 1–5) while the last question has 4 options (scored 1–4). The total score ranges form 3–14 with scores 3–8 indicating poor support, 9–11 indicating moderate support and 12–14 indicating strong support. It has been validated and used in Nigeria [16].
4. *Adverse childhood experience* was measured with the Adverse Childhood Experience Scale (ACE). The ACE is a 10-item questionnaire, which measures childhood experience of physical, emotional, sexual abuse and family dysfunction. It is scored based on Yes or No response with yes scored 1 and No scored 0. A score of 4 and above indicates high likelihood of adverse childhood experiences. It has been validated and used in Nigeria [17, 18].
5. *Depression and Suicidality* were assessed using the specific modules of the Child version of the Mini International Neuropsychiatric Interview for Children and Adolescents (MINI-KID). The MINI-KID is a pediatric form of the MINI, it is a structured diagnostic instrument designed to assess for the DSM-IV diagnostic categories. It is brief and can be administered within 10–15 min.
  The major depressive module contains questions labelled A1–A6 with a YES or NO response. Question labelled A3 is sub divided into questions A3a to A3g for both current (within 2 weeks) and past (past episode) major depressive episodes, respectively. A diagnosis of major depressive episode was made when 5 or more answers in A1–A3 coded YES and A4 coded YES. The questions were also structured to allow for specification into current (within 2 weeks) or past episode [19].
  The suicidality module was labelled questions B1–B13 with a YES or NO Response to each of the questions; questions B1–12 were events in the past month while B13 was event in lifetime. A NO response was ascribed a zero point in all the questions, while a variable point was ascribed for a YES response in each question; a Total score of 1–8 points indicate low suicidality, 9–16 points indicates moderate suicidality while 17 and above points indicates high suicidality, Also a YES response to any of the question except B1 indicated suicidality [19]. The scoring frame which reported Suicidality as either present (YES) or absent (NO) and the frame which showed the degree of severity (low, moderate and high) was used in this study.

### Procedure

Ethical approval; Ethical approval was obtained from the research ethics committees of the LASUTH and NIMER. A consent form, duly signed was taken from the parent/caregiver while a verbal assent from the adolescents aged less than 18 years. For the adolescents aged 18–19 years, an informed consent was taken after they had been adequately informed about the nature, extent, purpose and benefits of the study.

Sample size: the calculated sample size of 201 has 80% power to detect significant difference at 0.05 with standard normal deviation set at 1.96.

Data collection: A convenience sampling technique was used in selection of the adolescents. All adolescents presenting to the clinic meeting the eligibility criteria were recruited into the study. After consent and assent had been obtained, recruited adolescents were given the questionnaire to complete in a private consulting room before the clinician attended them to. While they self-completed the socio-demographic questionnaire, OSLO-3 and the ACE, the researcher then administered the Depression and Suicidality modules of the MINI-KID. Adolescents requiring help in understanding some aspects of the first part of the questionnaire were helped by the researcher who explained the question items. Only one researcher administered the MINI-KID to all the participants to ensure high reliability and remove inter-rater bias. The total length of interview averaged about 40 min per participants. The CD4 count value was extracted from the laboratory results in the patient record, the difference between the CD4 count done a year prior to the study and their most resent values not older than a month from the time of the study was calculated and recorded. Fifteen adolescents declined participation, but none discontinued during the study. Recruitment stopped when the goal of calculated sample size of two hundred and one participants was met. Those meeting the diagnosis of depression and/or suicidal behaviour were further assessed clinically and referred to a mental health facility of their choice.

### Statistical analysis

The Statistical Package for Social Sciences (IBM-SPSS) version 20 was used to analyze all collated data. Descriptive statistics was generated for relevant socio-demographic and clinical variables in the participants. The association between two categorical variables was determined using a Chi square while the T-test was used to compare mean values of continuous variables between the groups. Spearman correlation was used to determine the correlation between the dependent variables, while Binary logistic regression was used to determine the variables that were independently associated with depression or suicidal behavior in the stated population.

## Result

The mean age of the participants was 13.88 (± 2.53) years (Table 1). There were 95 participants in the early adolescents (10–13 years) group, 81 in the middle group (14–16 years) and 25 in the late adolescents (17–19 years). There were 124 (61.7%) male and 77 (38.2%) female participants. Majority (75.6%) of the participants were in secondary school, 48.3% (n = 97) of the adolescents had both parents as their primary care giver while 30.9% (n = 62) of them had either of the parents as their primary care givers. Fifty-seven (28.4%) participants had high adverse childhood events, 73.6% (n = 148) and 57.2% (n = 115) had history of physical and emotional abuse respectively. Seventy-five (37.3%) participants had poor social support. Table 1.

**Table 1:** Frequency table of independent variables n = 201

Variables	Frequency	Percentage	Mean (sd)
Age (in years)			13.88 (± 2.53)
Male	124	61.7	
Female	77	38.3	
Religion			
Christianity	158	78.6	
Islam	43	21.4	
Class in school			
Primary	38	18.9	
Junior secondary school	76	37.8	
Senior secondary school	76	37.8	
Post-secondary school	11	5.5	
Primary care giver			
Both parent	97	48.3	
Mother only	43	21.4	
Father only	19	9.5	
Others	42	20.9	
Family type			
Monogamous	137	68.2	
Polygamous	64	31.2	
Oslo score			
Poor social support	75	37.3	
Moderate social support	86	42.8	
Strong social support	40	19.9	
Adverse childhood event score (ACE)			
High adverse event (≥ 4)	57	28.4	
Low adverse event (< 4)	144	71.6	
Physical abuse			
Yes	148	73.6	
No	53	26.4	
Emotional abuse			
Yes	115	57.2	
No	86	42.8	
Route of infection			
At birth	175	87.0	
Sharp object	15	7.5	
Blood transfusion	11	5.5	
Hospital admission due to HIV			
Yes	46	22.9	
No	155	77.1	
Family member loss due to HIV			
Yes	48	23.9	
No	153	76.1	
Careful to disclose status			
Yes	177	88.1	
No	24	11.9	
Change CD4 count			
Increased	82	40.8	
Decreased	63	31.3	
Missing/incomplete data	56	27.9	

### Prevalence of depression and suicidality

According to the MINI Kid depression and suicidality modules, 34 (16.9%) and 90 (44.8%) of the participants were considered to have current and lifetime prevalence of major depressive episode respectively. Also, the current and lifetime rates for suicidal ideation were 14.9% (n = 30) and 33.3% (n = 67) respectively. For suicidal intent it was 8.5% (n = 17) and 17.5% (n = 35) respectively and for suicidal attempts the current and lifetime rates were 1.0% (n = 2) and 1.5% (n = 3) respectively. There were 35.3% (n = 71) participants meeting the criteria for suicidality. Out of this figure there were 50.7% (n = 36) ranked as having low suicidality, 31.0% (n = 22) having moderate suicidality and 18.3% (n = 13) having high suicidality.

### Correlates of major depressive episode and suicidality among adolescents with HIV infection

The adolescents were dichotomized into depressed and non-depressed groups, for both lifetime and current depressive disorders. In bivariate analysis the variables that were initially significantly associated with current depressive episode were female gender (P = 0.021), high ACE (p = 0.008) and decreased CD4 count (0.020), while for lifetime depressive episodes were poor social support (p = 0.029), high ACE (p = 0.042), physical abuse (p = 0.013), contacting HIV through means other than birth (p = 0.024) and disclosure of status (p = 0.022). See the full details in Table 2.

**Table 2:** Correlates of major depressive disorder

Variables	Current major depressive disorder					Life time major depressive disorder				
	Yes	No	Total	x^2^	p	Yes	No	Total	x^2^	p
Gender										
Male	15 (12.1)	109 (87.9)	124	5.348	*0.021*	50 (40.3)	74 (59.7)	124	2.596	0.107
Female	19 (24.7)	58 (75.3)	77			40 (51.9)	37 (49.1)	77		
Primary care giver										
Both parent	15 (15.5)	82 (84.5)	97	0.281	0.596	39 (40.2)	58 (59.8)	97	1.583	0.208
With one parent/others	19 (18.3)	85(81.7)	104			51 (49.0)	53 (51.0)	104		
Family type										
Monogamous	22 (16.1)	115 (83.9)	137	0.225	0.635	59 (43.1)	78 (56.9)	137	0.509	0.476
Polygamous	12 (18.8)	52 (81.2)	64			31 (48.4)	33 (51.6)	64		
Oslo score										
Poor social support	15 (20.0)	60 (80.0)	75	3.205	0.201	40 (53.3)	35 (46.7)	75	7.060	*0.029*
Moderate social support	16 (18.6)	70 (81.4)	86			39 (45.3)	47 (54.7)	86		
Strong social support	3 (7.5)	37 (92.5)	40			11 (27.5)	29 (72.5)	40		
ACE score										
High	16 (28.1)	41 (71.9)	67	7.044	*0.008*	32 (56.1)	25 (43.9)	67	4.155	*0.042*
Low	18 (12.5)	26 (87.5)	144			58 (40.3)	86 (59.7)	144		
Physical abuse										
Yes	27 (18.2)	121 (81.8)	148	0.704	0.401	74 (50.0)	74 (50.0)	148	*6.194*	*0.013*
No	7 (13.2)	46 (86.8)	53				16 (30.2)	37 (69.8)	53	
Emotional abuse										
Yes	23 (20.0)	92 (80.0)	115	1.82	0.177	58 (50.4)	57 (49.6)	115	3.481	0.062
No	11 (12.8)	75 (87.2)	86			32 (37.2)	54 (62.7)	86		
Route of infection										
Birth	28 (16.0)	147 (84.0)	175	0.807	0.369	73 (41.7)	102 (58.3)	175	5.129	*0.024*
Other	6 (23.1)	20 (76.9)	26			17 (65.4)	9 (34.6)	26		
Family member loss due to HIV										
Yes	7 (14.6)	41 (85.4)	48	0.244	0.621	27 (56.2)	21 (43.8)	48	3.357	0.067
No	27 (17.6)	126 (82.4)	153			63 (41.2)	90 (58.8)	153		
Careful to disclose status										
Yes	28 (15.8)	149 (84.2)	177	1.267	0.260	74 (41.8)	103 (58.2)	177	5.282	*0.022*
No	6 (25.0)	18 (75.0)	24			16 (66.7)	8 (33.3)	24		
Change CD4 count N = 145										
Increased	7 (8.5)	75 (91.5)	82	5.388	*0.020*	32 (39.0)	50 (61.0)	82	0.431	0.511
Decreased	14 (22.2)	49 (77.8)	63			28 (44.4)	35 (55.6)	63		

Significant values in italic

x^2^, chi-square; p, significant value

Similarly, participants were also dichotomized into those with suicidality and those without suicidality, ACE score (p = 0.025), physical abuse (p = 0.003), emotional abuse (p = 0.005) and disclosure of HIV status (p = 0.040) were the only variable that were statistically associated during the bivariate analysis. Table 3

**Table 3:** Correlates of suicidality

Variables	Suicidality			Statistics	
	Yes (n%)	No (n%)	Total	X^2^	p
Gender					
Male	38 (30.6)	86 (69.4)	124	3.101	0.078
Female	33 (42.9)	44 (57.1)	77		
Primary care giver					
Both parent	31 (32.0)	66 (68.0)	97	0.929	0.335
With one parent/others	40 (38.5)	64 (61.5)	104		
Family type					
Monogamous	44 (32.1)	93 (67.9)	137	1.936	0.184
Polygamous	27 (42.2)	37 (57.8)	64		
Oslo score					
Poor social support	29 (38.7)	46 (61.3)	*75*	1.458	0.482
Moderate social support	31 (36.0)	55 (64.0)	86		
Strong social support	11 (36.0)	29 (72.5)	40		
ACE score					
High	27 (47.4)	30 (52.6)	57	5.053	*0.025*
Low	44 (30.6)	100 (69.4)	144		
Physical abuse					
Yes	61 (41.2)	87 (58.8)	148	8.531	*0.003*
No	10 (18.9)	43 (81.1)	53		
Emotional abuse					
Yes	50 (43.5)	65 (56.5)	115	7.824	*0.005*
No	21 (24.4)	65 (76.5)	86		
Route of infection					
Birth	60 (34.3)	115 (65.7)	175	0.638	0.425
Other	11 (42.3)	15 (57.7)	26		
Family member loss due to HIV					
Yes	19 (39.6)	29(60.4)	48	0.501	0.479
No	52 (34.0)	101(66.0)	*15*		
Careful to disclosure status					
Yes	58 (32.8)	119 (67.2)	177	4.236	*0.040*
No	13 (54.2)	11(45.8)			
CD4 count N^*^ = 145					
Increased	23 (28.0)	59 (72.0)	82	2.783	0.095
Decreased	26 (41.3)	37 (58.7)	63		

x^2^, chi-square; p, significant value

Significant values in italics (p ≤ 0.05)

All the variables that were significantly associated with either depression and suicidality were entered into a logistic regression, Gender (Wald 9.084, OR = 0.280, 95% CL 0.122–0.641), ACE (Wald 8.362, OR = 3.363, 95% CL 1.478–7.651) and Change in CD4 (Wald 6.987, OR = 0.240, 95% CL 0.083–0.692) count were the only variables that emerged in the final regression model to be independently associated with current major depressive episode. Total OSLO score (Wald 8.805, OR = 0.516, 95% CL 0.198–0.929) and route of contact (Wald 7.807, OR = 0.0.263, 95% CL 0.103–0.671) were the only variables that emerged in the final regression model to be independently associated with lifetime major depressive episode. While none of the variables was independently associated with suicidality. Table 4.

**Table 4:** Binary logistic regression of associated variables

95% confidence interval								
Variables	B	SE	WALD	df	P	OR	Lower	Upper
Current major depressive disorder								
Gender (male)	− 1.273	0.422	9.085	1	*0.003*	0.280	0.122	0.641
High ace score	1.213	0.419	8.362	1	*0.004*	3.363	1.478	7.651
Change in CD4	− 1.426	0.539	6.987	1	*0.008*	0.240	0.083	0.692
Constant	0.891	0.423	4.447	1	0.035	0.410		
Lifetime major depressive disorder								
Physical abuse	0.505	0.386	1.713	1	0.191	1.657	0.778	3.529
Total oslo score	− 0.217	0.073	8.805	1	*0.003*	0.516	0.198	0.929
Careful To disclose	− 0.662	0.490	1.829	1	0.176	0.516	0.198	1.346
High ace score	0.381	0.355	1.131	1	0.283	1.464	0.730	2.938
Contact route	− 1.335	0.478	7.807	1	*0.005*	0.263	0.103	0.671
Constant	3.100	0.999	9.623	1	0.002	22.187		
Suicidality								
Careful to disclose	− 0.544	0.463	1.380	1	0.240	0.581	0.234	1.438
Physical abuse	0.710	0.453	2.455	1	0.117	2.034	0.837	4.945
Emotional abuse	0.357	0.381	0.876	1	0.349	1.429	0.677	3.017
High ace score	0.219	0.363	0.365	1	0.546	1.245	0.612	2.534
Constant	− 1.050	0.576	3.317	1	0.069	0.350		

B, coefficient for the constant (intercept); SE, Standard Error; WALD, Wald chi-square test; OR, Odd Ratio

P, significant P value in italic font

### Correlation between major depressive episode and suicidality

The spearman’s rho analysis showed a positive correlation between major depression and suicidality. Suicidality however had a relatively stronger correlation with lifetime major depressive episodes (r = 0.600, p < 0.01) compared to current depressive episode (r = 0.410, p < 0.01). There was also a positive correlate between current and lifetime major depressive episode (r = 0.501, p < 0.01). Table 5.

**Table 5:** Spearman’s rho correlation between major depressive episode and suicidality

		Current depressive episode	Suicidality
Lifetime depression episode			
Correlation coefficient		0.501^a^	0.600^a^
Sig. (2-tailed)		0.000	0.000
Current depressive episode			
Correlation coefficient		1.000	0.410^a^
Sig. (2-tailed)			0.000

^a^Correlation is significant at the 0.01 level (2-tailed)

## Discussion

Adolescents with HIV infection are confronted with challenges which makes them more prone to co-morbid mental illness such as depression and suicidality [20]. This study evaluated depression and suicidality in adolescents within the age range of 10–19 years attending two outpatient HIV clinics in Lagos state, Nigeria.

The prevalence of 44.8% reported for lifetime major depressive episode among adolescents with HIV in the current study is comparable to the rate of 40.8% [8] reported among adolescents with HIV/AIDS in Uganda and 39.9% [21] reported in a Nigerian study. In contrast, a lower rate of 21% was reported among HIV-infected patients in Cameroon [22]. This disparity may be because the Cameroon study was a community study as compared to the current study, which was a clinical study. Also, a prevalence of 16.9% for current major depressive episode reported in the current study among adolescents with HIV was similar to the prevalence of 18.9% and 20% reported among adolescents with HIV/AIDS in Malawi [7, 23]. This was however higher than the prevalence of 6.9% reported among general population of adolescents in Nigeria [6], thus emphasizing the previous report that adolescents with HIV infection were more prone to having depression [4, 20].

Previous study carried out among individuals with HIV in Benin city Nigeria to determine the level of suicidality, reported a prevalence of 34.7% [24]. In consonance with this finding, the current study found a prevalence of 35.3% for suicidality among adolescents with HIV. The psychological burden of being HIV positive and the need for a lifelong treatment may have accounted for the high prevalence of suicidality found among this group of individuals.

### Correlates of major depressive disorder

The female gender was found to be significantly associated with current major depressive episode. This is in keeping with previous studies that reported a strong association between depression and female gender [23, 25]. Reasons for this gender difference had been described as multifactorial, adolescence girls have been reported to be more likely than boys to ruminate more on distressing situations rather than taking actions to relieve them thus making them more prone to depression. Similarly, girls compared to boys are faced with more diverse social pressure such gender role conformity, abuse, poverty which make them more prone to depression [26].

Just as it has been reported in previous Nigerian study [9], adolescents living with HIV in the current study who had history of poor social support were more prone to having a lifetime major depressive episode compared to those with moderate to strong social support. A similar finding has also been documented in the United States of America [27]. This is not surprising; good social support has been reported to create a sense of belonging in individuals, and as such make them feel loved and cared for, and help fulfill their emotional needs. Social support helps reduce stigma and hopelessness, thus prevents and or reduces the psychosocial burden related to HIV infection [28, 29].

Adverse childhood event has been identified as a risk factor for depression in adolescents with HIV infection [29]. In keeping with previous studies, the current study also shows that participants with high adverse childhood experience were more likely to be depressed (lifetime and current major depressive episode) when compared to those with low adverse childhood experience. This may be attributed to the fact that adverse childhood experiences may predispose to low self-esteem, worthlessness and subsequently, depression [29]. It is also evident that child abuse is a serious global problem that is deeply rooted in cultural, economic, religious and social practices, and occurs in a variety of ways and settings. According to the National Child Abuse and Neglect Data System (NCANDA), abuse in children has different physical and psychological consequences [30]. Nigerian children and adolescents are victims of physical and emotional abuse, majority of which are underreported [31]. It is not surprising therefore that physical abuse was found to be associated with lifetime major depressive episode in the current study. This discovery is supported by finding from an earlier study carried out in South Africa which reported that individuals with history of physical abuse in childhood were more likely to develop major depressive episode compared to those who had no history of such experience [25]. The reason may be linked to the psychological impact that may develop from such experience; physical abuse may lower the self-esteem of a child and further affect their ability to cope with other challenges such as HIV infections. The study in South Africa also reported an association between emotional abuse with current major depressive episode [32]. However, in the current study emotional abuse was not significantly associated with either lifetime or current depressive disorder. Methodological differences may have accounted for this, as the South African study had a larger sample size and was conducted in a rural area, unlike the current study that was a hospital-based study in a cosmopolitan city.

Adolescents who contacted HIV through non-vertical route such as sharing of sharp objects and blood transfusion were also more likely to have lifetime major depressive episode than those who contacted it through vertical transmission. Although findings on the relationship between disclosure of HIV status and depression in previous studies have been inconsistent, the significant association found between disclosure of HIV status to others and lifetime major depressive episode in this study is in consonance with findings from some previous studies [27, 33].

In this study, Adolescents who were careful about the disclosure of their HIV status to others were less likely to have lifetime depressive episode compared to those who have disclosed their HIV status. Contrary to these findings, some studies suggested that disclosure of HIV status was protective to the individual, they reported that individuals who tell others about their illness tend to get more support and feel less burdened about their status compared to those who were careful to disclose their status [28, 34, 35]. However, in a randomized controlled interventional study carried out among HIV-affected adolescents in United States of America, there was no relationship between disclosure of HIV status and major depressive episode [36]. These disparities may be due to methodological differences, cofounder such as stigma and social disparities. For these reasons, further study will be necessary to ascertain this relationship.

An indirect relationship has been shown to exist between CD4 count and depression. CD4 count could indirectly be linked to depression and vice versa. Individuals with low CD4 count tend to have reduced immunity, higher incidence of opportunistic infection and hospital admission. These factors lead to deterioration in their overall quality of life and a subsequent increase vulnerability to depression [11–13]. Inversely, a low CD4 count can be an indicator of untreated depression [37]. These reasons may explain why there was a significant relationship between reduction in CD4 count and current major depressive episode in the current study. This finding further suggested the need for regular monitoring of CD4 level during the treatment of adolescents living with HIV.

### Correlates of suicidality

Adverse childhood event has been shown to be associated with increased rate of suicidality. This relationship has been consistently demonstrated in previous studies [15, 38–40]. The positive relationship between high Adverse Childhood Events (ACE) scores and suicidality reported in the current study is therefore not surprising. Similarly, Physical and emotional abuse were significantly associated with suicidality just as it was reported in previous studies [32, 41].

The National Clearinghouse on Child Abuse and Neglect Information defines emotional abuse as; “acts or omissions by the parents or other caregivers that have caused, or could cause serious behavioral, cognitive, emotional or mental disorders” [42]. Abuse can thus be seen as any attitude of a caregiver, which interferes with a child’s mental health, and social development. It attacks the child self-concept and esteem, making the child perceive him/herself as unworthy, worthless and incapable. Frequent abuse may lead to social withdrawal, negative self-concept, poor self-esteem, hopelessness and a resultant suicidality [43, 44] Similar to previous Nigerian study, the current study shows that adolescents who have disclosed their HIV status were found to have a significantly high rate of suicidality compared to those who were careful about the disclosure of their HIV status [45]. People whose HIV status was known to others experience discriminations which later triggers suicidality in them [46].

This study revealed a positive correlation between suicidality and depression among adolescents living with HIV infection. It showed a stronger positive correlation between suicidality and lifetime depression compared to suicidality and current episode. However further study will be necessary to determine causality, because due the cross-sectional nature of this study it could not be ascertained if depression lead to suicidality or vice versa. Another limitation of this study is the general methodological problems related to self-report assessment based on participants’ declarations, which may be affected by a social desirability bias.

## Conclusions

The findings from this study support reports in previous literature and gives further evidence that in providing care to adolescents with HIV infection, a multifaceted approach to management is necessary. As such, adolescents living with HIV infection should be provided with treatment options that meet their bio-psycho-social needs through the use of both medications and psychosocial treatments. It also points to the importance for the campaign against child abuse, and the need for clinicians to re-emphasize the importance of social support by the care giver to minimize the likelihood of these adolescents developing depression and suicidality.

## Data Availability

The datasets generated and analyzed during the current study are available from the corresponding author on request.
